# Mitochondrial Dysfunction in MASLD: Evidence in Dietary Models and Potential Therapeutic Interventions

**DOI:** 10.1155/bri/6641383

**Published:** 2026-05-19

**Authors:** Elda Cristina Villaseñor-Tapia, Edgar Rubén Mendieta-Condado, David Alejandro Curiel-Pedraza, Edwin Estefan Reza-Zaldívar, Ana Laura Márquez-Aguirre

**Affiliations:** ^1^ Unidad de Biotecnología Médica Farmacéutica, Centro de Investigación y Asistencia en Tecnología y Diseño Del Estado de Jalisco A.C., Jalisco, Mexico, ciatej.net.mx; ^2^ Servicios de Salud Jalisco, Jalisco, Mexico; ^3^ Escuela de Medicina y Ciencias de La Salud, Tecnológico de Monterrey, Monterrey, Mexico, tec.mx

**Keywords:** lipid metabolism, metabolic dysfunction–associated steatotic liver disease (MASLD), mitochondrial dysfunction

## Abstract

Metabolic dysfunction–associated steatotic liver disease (MASLD) is defined as the presence of excess triglyceride storage in the liver in the presence of at least one cardiometabolic risk factor. This term highlights the connection between fatty liver and metabolic dysfunction. Dietary factors, such as excessive consumption of saturated fats and sugar, contribute significantly to the accumulation of lipids in organs not specialized for fat storage, such as the liver. Hepatic lipid accumulation initiates dynamic changes in mitochondrial function and promotes the development and progression of MASLD. High‐fat, high‐fructose diet models have provided crucial insights into how nutritional factors induce mitochondrial dysfunction, which is characterized by impaired fatty acid oxidation, excessive generation of reactive oxygen species, and damage to mitochondrial DNA. Lifestyle modifications, including dietary adjustments such as calorie restriction and weight loss, are essential for the early prevention and long‐term treatment of MASLD. However, in recent years, several pharmacological options have emerged for the treatment of MASLD, primarily for the management of its comorbidities. This review explores the mechanisms of mitochondrial dysfunction promoted by the accumulation of hepatic lipids, analyzes the evidence of mitochondrial alterations in the liver of dietary models, and summarizes some of the main therapeutic interventions for MASLD and their effects on mitochondrial function.

## 1. Introduction

Metabolic dysfunction–associated steatotic liver disease (MASLD) is recognized as the most common cause of chronic liver disease around the world, and the prevalence exceeding 38% of the global adult population [1]. The term MASLD emphasizes the metabolic disturbances linked to fatty liver disease and its strong association with cardiometabolic risk factors (e.g., obesity, type 2 diabetes [T2D]) [2]. The EASLEASD‐EASO Clinical Practice Management Guidelines for MASLD classify the pathological spectrum of MASLD, ranging from fatty liver (steatosis) to metabolic dysfunction–associated steatohepatitis (MASH), fibrosis, and cirrhosis [3]. These pathophysiological changes range from mild to severe. In the preliminary phase (fatty liver), lipoprotein metabolism is disrupted as a result of decreased lipid uptake by hepatocytes, increased fatty acid synthesis, and reduced fatty acid oxidation. This lipid accumulation makes the liver more vulnerable, and MASLD progresses to MASH. MASH is characterized by hepatic inflammation, oxidative stress, apoptosis, and hepatocyte necrosis. If chronic hepatic stimulation from fat accumulation and inflammation persists, MASH progresses to fibrosis and cirrhosis [4, 5]

Mitochondria are found in significant numbers in hepatocytes and play a crucial role in metabolism, as they constitute the principal site for oxidative phosphorylation (OXPHOS) and fatty acid β‐oxidation (FAO), leading to ATP synthesis. Due to their role in FAO, lipogenesis, and gluconeogenesis, mitochondria are involved in the pathogenesis of MASLD. Dysfunctional mitochondria not only fail to oxidize fatty acids efficiently but also produce excess of reactive oxygen species (ROS), contributing to hepatocyte injury, inflammation, and fibrosis [6, 7].

The importance of dietary models in studying these mechanisms cannot be overstated, since they are the tool to study how saturation in lipid degradation produces a permanent inflammatory effect and a general condition known as lipotoxicity, partly produced by a substantial increase in oxidative stress. Nutritional factors like saturated fats and sugar consumed in excess are major contributors to metabolic diseases. Dietary models of MASLD have provided critical insights into how nutritional factors disrupt mitochondrial homeostasis, highlighting mitochondrial dysfunction as a key feature of human steatohepatitis [6, 7].

Several studies have shown that improving hepatic mitochondrial function through pharmacological or non‐pharmacological treatments can reduce the pathological characteristics of MASLD [8]. This review explores the mechanisms of mitochondrial dysfunction promoted by the accumulation of hepatic lipids, analyzes the evidence of mitochondrial alterations in the liver of dietary models, and summarizes some of the main therapeutic interventions for MASLD and their effects on mitochondrial function.

## 2. Lipid Metabolism in the Liver and the Role of the Mitochondria

The liver is a key metabolic organ responsible for lipid homeostasis, ensuring that lipids are properly synthesized, stored, metabolized, and distributed throughout the body for energy demands. Under normal physiological conditions, fatty acids are oxidized in the mitochondria to generate ATP or converted into ketone bodies, 3‐hydroxybutyrate, and acetoacetate, which are utilized as alternative sources of energy by extrahepatic organs, like the brain, when blood glucose levels are low [9].

Excessive lipid accumulation disrupts hepatic function and contributes to cell dysfunction, which subsequently leads to systemic metabolic dysregulation [10]. Lipids in the liver arise from three primary sources: (1) Circulating free fatty acids (FFAs) from the lipolysis of triglycerides (TG) in adipose tissue (approximately 60%) that are taken up by hepatocytes via specific transporters, such as fatty acid transport proteins (FATP) —typically FATP‐2 and ‐5; (2) dietary FFAs (amounting to about 15%) contained as TG within ApoE‐enriched chylomicrons remnants; and (3) FFAs synthesized (roughly 25%) from dietary carbohydrates through *de novo* lipogenesis (DNL), and regulated by the transcription factors such as sterol regulatory element‐binding protein 1c (SREBP‐1c) and carbohydrate‐responsive element‐binding protein (ChREBP) [11]. Once inside hepatocytes, fatty acids are esterified into TG and stored as lipid droplets or packaged into very‐low‐density lipoproteins (VLDL) for export. Alternatively, they can undergo β‐oxidation in mitochondria [11]. The dysregulation of one or more of these pathways, including DNL, VLDL production, decreased hepatic FAO, exacerbation of lipolysis in adipose tissue, and alterations of insulin production, is related to hepatic steatosis development [12–14].

At the cellular level, FAO occurs predominantly in mitochondria; long‐chain fatty acids are transported into mitochondria by the L‐carnitine shuttle system, primarily regulated by carnitine palmitoyltransferase 1 (CPT‐1). Inside mitochondria, these fatty acids undergo β‐oxidation to generate acetyl‐CoA, which fuels the tricarboxylic acid cycle (TCA) and ketogenesis. Additionally, mitochondria interact closely with lipid droplets to facilitate the transfer of fatty acids for oxidation and prevent the accumulation of toxic lipid intermediates. Excessive lipid influx overwhelms mitochondrial capacity, leading to fat accumulation in hepatocytes and promoting an increase in ROS production by mitochondrial FAO overload, which drives DNA, proteins, and lipids damage, perpetuating mitochondrial dysfunction, inflammation, and other metabolic dysfunctions [15, 16].

The main toxicity in these conditions is mediated by ceramides and diacylglycerols, and promotion of lipid‐induced cellular injury, including lysosomal dysfunction and endoplasmic reticulum (ER) stress, abnormal activation of intracellular signaling pathways, chronic inflammation, and hypoxia, ultimately leading to cell death [14]. Although the direct mechanisms responsible for fatty liver are still not fully elucidated, the association of mitochondrial abnormalities, including mitochondrial biogenesis, autophagy, mitophagy, fission, and fusion, with the decreased. The enhanced delivery of FFAs into the liver, along with the increased hepatic synthesis of lipids, is a key factor in the development and severity of MASLD [7].

## 3. Mechanisms of Mitochondrial Dysfunction in MASLD

Mitochondria are highly dynamic organelles that function as the central hubs for energy metabolism. Consequently, a key role of hepatic mitochondria is managing energy production. In healthy hepatocytes, mitochondria adapt to metabolic demands by adjusting their number, structure, and function through processes such as mitochondrial biogenesis, fission, fusion, and mitophagy.

**FIGURE 1:**
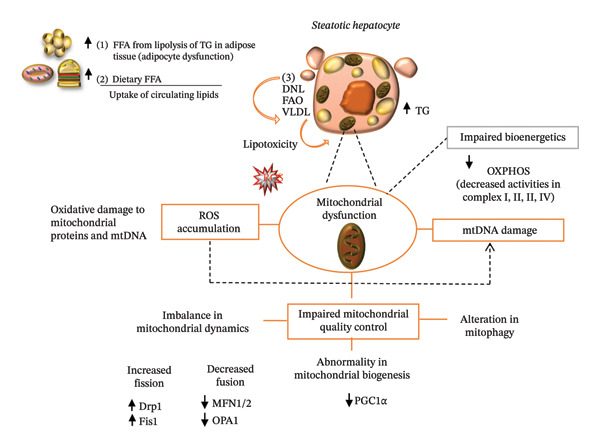
Mitochondrial dysfunction in steatotic hepatocytes. Lipids in the liver arise from three primary sources: (1) circulating free fatty acids (FFAs) derived from the lipolysis of triglycerides in adipose tissue; (2) dietary FFAs; and (3) FFAs synthesized from dietary carbohydrates through de novo lipogenesis (DNL). Mitochondria are essential organelles that play a central role in maintaining lipid homeostasis. However, mitochondrial abnormalities, including impaired mitochondrial biogenesis, autophagy, mitophagy, fission, and fusion, result in a reduced capacity to oxidize fatty acids, leading to an increased delivery and transport of FFAs to the liver.

In conditions such as MASLD, mitochondrial function is reduced, leading to various metabolic and cellular problems. Mitochondrial dysfunction is characterized by (1) decreased FAO, (2) alterations in mitochondrial quality control (including changes in mitochondrial dynamics, abnormal biogenesis, and altered mitophagy), (3) increased production of ROS, and (4) damage to mitochondrial DNA (mtDNA) [17] (Figure 1).

### 3.1. Impaired FAO

Disruption of FAO is one of the earliest mitochondrial abnormalities in MASLD. Mitochondrial β‐oxidation is the dominant oxidative pathway for the disposal of fatty acids under normal physiological conditions. It is primarily involved in the oxidation of short‐chain (< C8), medium‐chain (C8–C12), and long‐chain (C12–C20) fatty acids [9]. CPT‐1 (A, predominant liver isoform) is the rate‐limiting enzyme that facilitates the transport of long‐chain fatty acids into the mitochondrial matrix via transesterification reactions, followed by the intramitochondrial exchange of carnitine for CoA by CPT‐2 [18, 19].

In hepatic steatosis, mitochondrial dysfunction is often associated with the downregulation of peroxisome proliferator‐activated receptor alpha (PPARα), a master regulator of lipid metabolism. PPARα stimulates fatty acids import into the mitochondria by upregulating expression of CPT‐1 and CPT‐2, and the carnitine acyl‐carnitine translocase (CACT) [20, 21]. The PPARα impairment expression leads to reduced CPT‐1A activity, resulting in impaired fatty acid transport into the mitochondria and partial FAO [22, 23]

Another key molecule in the FAO is Malonyl‐CoA. This molecule is the substrate for fatty acid synthesis. It is an allosteric inhibitor of CPT‐1A, regulating that FAO does not co‐occur with synthesis, and thus of fatty acid entry to the mitochondria for β‐oxidation [24]. Dysregulation of malonyl‐CoA levels inhibits CPT‐1A activity and impairs FAO. A key enzyme involved in this regulation is the acetyl‐CoA carboxylase (ACC), the enzyme that catalyzes the carboxylation of acetyl‐CoA to form malonyl‐CoA. In conditions such as obesity, insulin resistance (IR), or MASLD, the ACC activity is upregulated, leading to increased malonyl‐CoA levels [25]. Interestingly, in lipogenic tissues such as the liver, the malonyl‐CoA produced by ACC1 in the hepatocyte cytosol may be primarily involved in the regulation of DNL, whereas the malonyl‐CoA produced by ACC2 in the mitochondria acts mainly in the regulation of mitochondrial FAO [26, 27]. The consequence is cytosolic lipid accumulation, where they are esterified into TG and other lipid species, contributing to lipid droplet formation in hepatocytes.

### 3.2. Mitochondrial Quality Control Failure

Mitochondrial quality control is a critical cellular process that ensures the integrity, functionality, and turnover of mitochondria. It involves a coordinated interplay of several processes, including (1) mitochondrial dynamics—fusion and fission of mitochondria, (2) mitochondrial biogenesis—generates new mitochondria from the existing ones, and (3) mitophagy—selectively eliminates defective mitochondria through fusion with lysosomes. All these processes restore the organelle’s homeostasis during energy deprivation or after a mitochondrial insult [17, 28].

#### 3.2.1. Mitochondrial Dynamics

Mitochondrial dynamics are essential for maintaining function, metabolic flexibility, and stress responses. This involves the mitochondrial processes of fusion and fission. Fusion is a process in which two or more mitochondria merge to form a larger organelle, whereas fission results in the creation of new mitochondria. Under physiological conditions, mitochondrial fusion is mediated by key proteins such as Mitofusin 1 (MFN1), Mitofusin 2 (MFN2), and Optic Atrophy 1 (OPA1), which facilitate the merging of mitochondrial membranes, allowing the exchange of contents such as mtDNA, proteins, and metabolites. This process ensures the complementation of damaged components, enhances OXPHOS efficiency, and supports metabolic flexibility, enabling hepatocytes to adapt to varying nutrient availability. On the other hand, mitochondrial fission, primarily regulated by Dynamin‐related protein 1 (Drp1) and its adaptor proteins (Fis1, MFF, MiD49, and MiD51), facilitates the division of mitochondria, enabling the segregation and removal of damaged or dysfunctional mitochondria through mitophagy, a process critically dependent on the PINK1‐Parkin pathway [16]. This process also facilitates mitochondrial redistribution to areas of high energy demand to preserve mitochondrial network health, in addition to regulating morphology and facilitating mitochondrial trafficking.

Numerous in vivo and in vitro studies emphasize that obesity and MASLD are associated with a disrupted balance between mitochondrial fusion and fission [16]. Excessive mitochondrial fission, often driven by hyperactivation of Drp1 due to high levels of FFA, leads to mitochondrial fragmentation, resulting in smaller, dysfunctional mitochondria that are inefficient at FAO and prone to generating ROS. The accumulation of fragmented, dysfunctional mitochondria promotes hepatocyte lipid accumulation, probably by reducing the capacity for β‐oxidation, leading to the accumulation of toxic lipid intermediates, which further impair mitochondrial function and insulin signaling [29]. High fat intake also impaired mitochondrial fusion, due to downregulation of MFN2, leading to fragmented mitochondrial networks, impairing the complementation of damaged components and reducing the efficiency of FAO [16, 30]. This is further exacerbated by the downregulation of OPA1, which disrupts inner mitochondrial membrane integrity and cristae structure, essential for optimal oxidative phosphorylation and FAO [31]. Additionally, impaired fusion and excessive fission disrupt mitochondrial function and suppress PPARα activity, which could lead to higher lipid droplet deposition [32]. This perpetuates oxidative stress, lipid peroxidation, and cellular damage.

#### 3.2.2. Mitochondrial Biogenesis

Mitochondrial biogenesis is the process through which cells increase their mitochondrial mass. It comprises mtDNA replication, the transcription of mtDNA and nuclear coding genes, and the translation and assembly of the OXPHOS complex [33]. Mitochondrial biogenesis is orchestrated by peroxisome proliferator‐activated receptor gamma coactivator‐1 (PGC‐1α), a master transcriptional activator of nuclear respiratory factors (NRF) 1 and 2, and estrogen‐related receptor (ERR) family, which promotes the expression of mitochondrial transcription factor A (TFAM) and other genes required for mtDNA replication, transcription, and the assembly of mitochondrial proteins [33]. PGC‐1α coactivates PPARα, which is a crucial regulator of FAO, thereby connecting mitochondrial biogenesis with lipid metabolism [28].

Additionally, there is evidence of regulation of mitochondrial biogenesis and turnover by silent information regulator 1 (SIRT1), particularly of PGC‐1α. SIRT1 deacetylates and activates PGC‐1α, promoting the transcription of nuclear genes. Accumulation of PGC‐1 α in the nucleus by SIRT1‐dependent deacetylation, increasing the PGC‐1 α’s transcriptional activity necessary for mitochondrial function and biogenesis [34].

#### 3.2.3. Mitophagy

Mitophagy is activated when mitochondrial damage exceeds the capabilities of other quantity and quality control methods, or when mitochondria are removed for cellular metabolic purposes. This process is dependent on two factors: Serin/threonine ‐PTEN‐induced putative kinase 1 (PINK1) and the E3 ubiquitin ligase. These proteins act in sensing the functional and health statuses of mitochondria, and mark damaged mitochondria for autophagic disposal [35].

Paradoxically, although SIRT1 has a role in mitochondrial biogenesis discussed above, it is also involved in the opposite process: the destruction of damaged or aged mitochondria through mitophagy. SIRT1’s regulation of general macroautophagy (an evolutionarily conserved pathway that consists of the process of lysosomal degradation and cellular component recycling) is well known and is broadly viewed as a cellular protective mechanism against stress and death insults [34].

Mitophagy involves the selective isolation and degradation of damaged mitochondria to maintain functional integrity and cellular homeostasis; it is a protective mechanism that enables the cell to avoid generating ROS. It stimulates lipid droplet breakdown and the release of fatty acids, which are then transported to healthy mitochondria for β‐oxidation and increased energy release [28, 33].

All these mitochondrial quality control mechanisms are essential for maintaining mitochondrial health and preventing the accumulation of damaged mitochondria that could negatively impact lipid metabolism.

### 3.3. Oxidative Stress and ROS Overproduction

Mitochondria are the primary intracellular source of ROS, generated naturally as byproducts of OXPHOS in the electron transport chain (ETC). During normal respiration, 1%–2% of electrons leak from complexes I and III, reacting with oxygen to form superoxide (O_2_^−^), which can be converted into hydrogen peroxide (H_2_O_2_) and, ultimately, hydroxyl radicals (HO^−^). Although the generation of ROS is part of core metabolism, its excessive production can lead to oxidative stress and disrupt lipid metabolism [36, 37]. Within mitochondria, this oxidative stress disrupts the integrity and function of mitochondrial membranes, leading to increased permeability, dissipation of the mitochondrial membrane potential (ΔΨm), and further ETC uncoupling. This not only amplifies ROS production in a feed‐forward manner but also impairs ATP synthesis, promoting energy failure in hepatocytes [38].

Mitochondrial damage caused by ROS leads to the release of proapoptotic factors such as cytochrome c, promoting hepatocyte apoptosis, which subsequently activates hepatic stellate cells and propagates inflammation in the liver [15, 17, 39, 40].

### 3.4. mtDNA Damage

mtDNA is particularly vulnerable to oxidative damage because of its proximity to the ETC, absence of protective histones, and limited DNA repair capabilities [41]. ROS‐induced damage to mtDNA leads to mutations, deletions, and impaired transcription of mitochondrial genes that encode critical components of the respiratory chain. Extensive mtDNA damage can exacerbate oxidative stress and destroy the mitochondrial respiratory chain and energy metabolism, thus contributing to the pathogenesis of liver diseases [17].

Such mtDNA alterations can further impair ETC function, resulting in decreased ATP production and exacerbated ROS generation, a self‐perpetuating loop of mitochondrial dysfunction. In MASLD, evidence of mtDNA damage has been observed in both experimental models and human patients [42]. An increased prevalence of cytochrome b mutations in liver samples from individuals with advanced MASH, correlating with severe oxidative injury and impaired metabolic function. Additionally, mtDNA fragments released into the cytosol or circulation can act as damage‐associated molecular patterns (DAMPs), activating innate immune responses via Toll‐like receptor 4/9 (TLR4/9) and inflammasomes [43]. This leads to further inflammation and liver injury.

Thus, mtDNA damage is not only a consequence but also a contributor to mitochondrial and hepatic dysfunction in MASLD, reinforcing the need to protect mitochondrial genomes as part of comprehensive therapeutic strategies.

## 4. Evidence of Mitochondrial Dysfunction in Dietary Models of MASLD

Owing to the multifactorial nature of MASLD, the combination of two or more inductions (dietary interventions, genetic manipulations, and/or administration of chemical substances) is a usual approach to better mimic human disease [44]. Dietary interventions in rodents are one of the most common models used in MASLD research. Experimental dietary models are usually attained through the provision of either excess calories from fat (45%–60%) or excess calories from refined carbohydrates (glucose, fructose) or excess calories from a combination of fat, cholesterol, and carbohydrates. These latter models emerge as the most balanced in terms of metabolic, histologic, and transcriptomic similarities to human MASLD [45]. Evidence of mitochondrial dysfunction has been documented in many of these diets (Table 1).

**TABLE 1:** Evidence of mitochondrial dysfunction in some rodent dietary models of MASLD.

Dietary interventions			Evidences on mitochondrial function	References
High‐fat diet	HF	Sprague‐Dawley rats liquid diet with 71% fat, 11% carbohydrates, and 18% protein	Abnormal mitochondria with degenerative changes, including rarefied matrix and loss of cristae	[47]
		3 weeks		
	HF	C57BL/6 mice	Mitochondrial ROS were increased, and the liver mitochondrial proteome was altered	[46]
		71% fat, 11% carbohydrates, and 18% protein		
		16 weeks		

High‐fat/high‐fructose diet	HF‐F	Sprague–Dawley rats	Lower mitochondrial oxidative capacity but significantly higher oxidative stress	[48]
		42% lipids, 20% protein, and 25% fructose		
		2 weeks		
	HFHFR	Wistar rats	Mitochondrial ROS production was increased	[49]
		35% lipids (1% soyabean oil + 8·5% lard + 17% palm oil + 8·5% cocoa butter) and 25% fructose	Defects in mitochondrial respiratory chain enzyme complexes	
		20 weeks	Changes in the content and/or the composition of mitochondrial membrane phospholipids	
	HF + Fr	Wistar rats	Mitochondrial ROS levels and lipid peroxidation were increased	[50]
		14.7% protein, 53.8% fat, and 29.8% carbohydrates	Lower mitochondrial state 3 respiration	
		25% fructose in drinking water		
		6 weeks		
	HF/HFr	C57BL/6NJ mice	Higher expression of proteins involved in OXPHOS	[51]
		25% kcal fat, 20% kcal protein, and 34.9% kcal fructose; (D19013101)		
		24 weeks		
	HF‐HFRU	C57BL/6 mice	Mitochondrial dysfunction and ER stress	[52]
		32% of energy as lard, 10% as soybean oil, and 32% of energy as fructose		
		12 weeks		

High‐fat/high‐fructose/high‐cholesterol diet	WD30	C57BL/6J mice	Hepatic mitochondrial changes appear to be adaptations to prevent overload of the respiratory chain	[53]
		40% fat, 15.5% protein, 44% carbohydrate		
		AIN‐76A WD (D12079B) glucose (18.1 g/L) and fructose (24 g/L) in drinking water		
		30 weeks		
	HFHC	C57BL/6N mice	Downregulation of key OXPHOS components (mt‐Nd4, mt‐Cytb, Uqcrq)	[54]
		High‐fat, high‐cholesterol, sugar‐free diet(#TP26304B) plus 10% fructose, glucose, or sucrose in drinking water		
		10 weeks		

*Note:* High‐fat diet = HFD, HF; high‐fructose diet = HFr, F, Fr, HFRU; WD = Western diet style.

Abbreviation: HC = high cholesterol.

Several studies examining the relationship between high‐fat diet (HFD) and MAFLD, with diverse biological mechanisms such as IR, impaired autophagy, gut microbiota imbalance, ER stress, oxidative stress, inflammation, apoptosis, and mitochondrial dysfunction [4]. Some of the earliest studies already reported alterations in mitochondrial structure and proteome [46, 47]. Later, to mimic the modern Western‐style diet (WD), the dietary models with high fat (HF) were supplemented with high fructose (HFr) and high cholesterol (HC). These studies demonstrate that HF/HFr feeding is more conducive to the development of liver steatosis and deleterious to glucose homeostasis than HF. Lower mitochondrial oxidative capacity but significantly higher oxidative stress was found in rats fed HF/HFr [48]. The major fatty acid composition of liver mitochondrial phospholipids was also altered after HF/HFr intake [49]. Besides, to get more insight into whether fructose or fat is more deleterious in both liver damage and mitochondrial dysfunction, in 2019, García‐Berumen compared the effects of HF, HFr, and their combination. They found that the HF plus Fr showed more severe damage in liver tissue, and mitochondria exhibited fully inhibited state 3 respiration, impaired complex I activity, and increased ROS generation [50]. More recent studies confirm that high consumption of both (HF/HFr) contributed to accelerate metabolic dysfunction during MASLD, and revealed the intimate relationship between DNL and altered mitochondrial dynamics [51–53]. Recently, a study by Li et al., comparing the different effects of fructose, glucose, and sucrose consumption in mice fed HF and HC diets (HFHC), showed that fructose intake (but not glucose or sucrose) aggravated liver damage and IR in these animals, and identified key signaling pathways, including specific suppression of OXPHOS [54].

In summary, dietary models of MASLD not only confirm the detrimental effects of overnutrition on mitochondria but also highlight the reversibility of such effects, emphasizing the therapeutic potential of dietary interventions. Targeting mitochondrial pathways through pharmacological or lifestyle interventions represents a promising therapeutic approach for the management of metabolic diseases [12, 55, 56].

## 5. Therapeutic Interventions for MASLD/MASH and Their Effects on Mitochondrial Function

Effective management of MASLD focuses on the alterations that drive disease progression. Non‐pharmacological therapies, such as dietary interventions and increased physical activity, improve MASLD. However, maintaining these changes in practice can be challenging, and various pharmacological options are still being investigated in both clinical and preclinical trials, primarily for MASH with liver fibrosis. It is worth noting that while some of these interventions are not specifically indicated for the treatment of MASLD (but rather for treating some of its comorbidities, such as obesity and T2D), and only a couple of them have already been approved for the treatment of MASH. Additionally, it should be noted that although mitochondria were not the primary target, there is evidence that many of these therapies also improve mitochondrial function (Table 2).

**TABLE 2:** Therapeutic interventions for MASLD/MASH and their effects on mitochondrial function.

Treatment	Categories	Therapeutics	Examples	Effects on mitochondrial function	References
Non‐pharmacological therapy	Lifestyle interventions	Diet	Mediterranean diet	Improves mitochondrial biogenesis	[58, 60]
			Caloric restriction diets	Activates antioxidant defenses	
			Intermittent fasting	Reduces ROS formation	
		Exercise	Aerobic exercise regimen	Prevents ER stress and improves hepatic beta‐oxidation	[66]
			Resistance training	Promotes increased auto/mitophagy and mitochondrial fusion	

Pharmacological therapy	Liver‐targeting molecules	THR‐β agonists	Resmetirom (Rezdiffra^∗^)	Increases mitochondrial FAO, respiratory capacity, and biogenesis. Decreases lipotoxicity, oxidative stress, and DNL	[69, 70]
		FGF‐21 analogs	Efruxifermin	Increases mitochondrial biogenesis, oxidative capacity, and lipid utilization	[54]
			Pegozafermin Pegbelfermin	Decreases oxidative stress and activates mitophagy/autophagy	
	Drugs for obesity and T2D with benefits in MASLD/MASH	GLP‐1R agonists	GLP1‐RAs (Semaglutide‐Wegovy^∗^)	Increases mitochondrial efficiency in skeletal muscle	[78–81]
			(Liraglutide)	Reduces hepatic DNL	
			Dual GLP‐1/GIP agonists (Tirzepatide)	Decreases lipotoxicity	
				Improves redox status and mitochondrial respiration	
		PPAR agonists	Dual agonists PPARγ/PPARα (Pioglitazone)	Increases FAO and decreases hepatic TG accumulation	[86]
			pan‐PPAR agonists (Lanifibranor)	Improves lipid flux for oxidation. Decreases lipotoxicity	
				Indirectly improves mitochondrial efficiency	
				Promotes mitochondrial biogenesis	
		SGLT‐2 inhibitors	Empagliflozin Dapagliflozin	Decreases ROS production	[92, 93]
				Upregulates mitochondrial biogenesis	
		Biguanides hypoglycemic drugs	Metformin	Increases FAO and mitochondrial biogenesis	[94, 95]
				Increases insulin sensitivity. Decreases DNL	
	Potential molecules for MASLD	MTAs	MitoQ (Mitoquinone)	Diminution of mitochondrial ROS production	[99, 100]
			SkQ1	Increase membrane potential and mitochondrial respiration	
			SS‐31(Elamipretide)		
			AntiOxBEN2		
		Synthetic SIRT1 activators	SRT2104	Increases mitochondrial biogenesis	[105, 106]
				Enhancing mitochondrial function Suppressing oxidative stress	
		DNL inhibitors	ACC inhibitors	Diminution of DNL and lipotoxicity	[108–110]
			FAS inhibitors	Indirect improvement of mitochondrial efficiency and diminution of ROS	

^∗^FDA‐approved drugs for the treatment of MASH.

### 5.1. Non‐Pharmacological Therapy

Lifestyle modification remains the cornerstone of MASLD management and exerts direct effects on mitochondrial health. Some recommended dietary strategies include the Mediterranean diet (MedDiet), DASH diet, the flexitarian diet, intermittent fasting (IF), ketogenic diet, and diets with high‐fiber, low‐fructose, and low‐simple sugars [57, 58]. The MedDiet is the main clinical recommendation of EASL–EASD–EASO guidelines for non‐pharmacological intervention in MASLD [3]. This diet is characterized by a high intake of olive oil, vegetables, fruits, whole grains, legumes, nuts, fish, and seafood. It also limits saturated sugars, refined carbohydrates, saturated fats, and ultra‐processed foods [59]. The effects of the MedDiet on mitochondria are attributed to its bioactive compounds, which improve overall mitochondrial health by modulating oxidative phosphorylation, biogenesis, and mitophagy [60]. Moreover, caloric restriction promotes mitochondrial biogenesis, reduces ROS production, and improves mitochondrial efficiency by activating AMP‐activated protein kinase (AMPK) and SIRT1 pathways [61]. This is one of the most effective interventions for reversing MASLD. A 5%–10% reduction in body weight is associated with improvements in hepatic steatosis, while ≥ 10% is required to reverse fibrosis [62]. Furthermore, IF regimens (e.g., 16:8 time‐restricted feeding) improve mitochondrial dynamics and autophagy. IF increases PGC‐1α, stimulates mitophagy, and reduces hepatic lipid accumulation in rodent models [63].

It is known that the interventions involving both exercise and dietary modification are effective. However, several studies have reported that exercise alone (without dietary interventions or significant weight loss) is able to reduce liver steatosis in animal models or individuals with MASLD [64–66]. Regular physical activity enhances mitochondrial content and function via increased expression of PGC‐1α and SIRT1. Both aerobic and resistance training have been shown to reduce liver fat independently of weight loss [37]. Hence, exercise increases FAO capacity, improves insulin sensitivity, and reduces oxidative stress, making it a potent mitochondrial modulator.

### 5.2. Pharmacological Therapy

Several classes of targets in the research and development pipeline have shown highly promising therapeutic efficacy, including liver‐targeting molecules (drugs originally developed for the treatment of MAFLD that target the liver as the primary organ) and drugs originally developed for obesity and T2D that showed beneficial effects on liver parameters. In fact, the promising results obtained in patients with T2D regarding body weight, IR biomarkers, and liver damage spurred the development of specific trials in overweight and obese individuals without diabetes and in patients with MASLD or MASH [67, 68].

#### 5.2.1. Liver‐Targeting Molecules

##### 5.2.1.1. THR‐b Agonists

Thyroid hormone (THR) receptors are located in the cell nucleus and exhibit variable tissue expression. The beta isoform (THR‐β) is highly expressed in the liver. Activation of THR‐β leads to a reduction in DNL, promotes FAO, modulates mitophagy and mitochondrial biogenesis, and may exert direct anti‐inflammatory and antifibrotic effects on liver tissue [69–71]. Although several compounds within this class are being studied, resmetirom is the most advanced drug in clinical trials, having reached the Phase 3 stage (MAESTRO‐NAFLD 1 MAESTRO‐OLE, MAESTRO‐NASH, MAESTRO‐NASH‐OUTCOMES) [67]. Furthermore, in 2024, the U.S. Food and Drug Administration (FDA)‐approved Rezdiffra (resmetirom) for the treatment of adults with MASH with moderate to advanced liver fibrosis. This represents a significant advance in targeted drug therapy for MASH as the first FDA‐approved treatment [72]. This drug is revolutionizing the field of treatment because it effectively resolves MASH and has thus far proven to be safe and well tolerated.

##### 5.2.1.2. FGF‐21 Analogs

Fibroblast growth factor 21 (FGF‐21) is a pleotropic liver‐derived hormone that has important roles in regulating glucose and lipid metabolism, energy homeostasis, and insulin sensitivity. In the liver, FGF21 modulates several intracellular pathways to protect hepatocytes against stress and cell death. For example, it increases the capacity of antioxidant pathways via activation of the nuclear factor erythroid‐2‐related factor 2 (Nrf2) and increases autophagy pathways, including lysosomal biogénesis [73, 74]. In recent years, FGF‐21 analogs and mimetics have progressed through the phases of clinical trials in patients with MASH [75, 76]. The first molecule to report Phase 2 results was pegbelfermin, but it did not show improvement in liver fibrosis (FALCON 1, FALCON 2). However, efruxifermin and pegozafermin have shown improvements in fibrosis over placebo in Phase 2b clinical trials (HARMONY, ENLIVEN), and are being moved to Phase 3 (SYNCHRONY, ENLIGHTEN) [67, 77].

#### 5.2.2. Drugs for Obesity and T2D With Benefits in MASLD/MASH

##### 5.2.2.1. GLP‐1 Receptor Agonists

Glucagon‐like peptide‐1 receptor agonists (GLP‐1RAs) are synthetic analogs of the endogenous GLP‐1 peptide. These agents act by modulating the satiety center in the brain, inhibiting glucagon secretion from pancreatic α‐cells, and stimulating insulin production from β‐cells through activation of the GLP‐1R [78–81]. Although they initially entered the market as glucose‐lowering drugs and some of them are now approved for the pharmacological treatment of both T2D and obesity, these agonists are a promising treatment option in MASLD, effectively achieving MASH resolution [67, 82]. In fact, recently, in 2025, the FDA made Wegovy (semaglutide) the first GLP‐1RA approved for MASH.

GLP‐1RAs have shown beneficial effects on multiple pathways, including insulin sensitivity, inflammation, oxidative stress, autophagy, DNL, and antifibrotic effects [83, 84]. Therefore, additional molecules capable of activating multiple incretin and hormone receptors have been developed (e.g., tirzepatide—an agonist for both glucose‐dependent insulinotropic polypeptide (GIP) and GLP‐1R, or retatrutide—a triple hormone agonist targeting the GIP, GLP‐1, and glucagon receptors [67, 85].

##### 5.2.2.2. PPAR Agonists

PPARs are nuclear receptors that regulate genes involved in lipid metabolism, inflammation, and mitochondrial biogenesis. Among them, PPAR‐α is particularly important for promoting FAO in the liver. Fibrates (e.g., gemfibrozil, fenofibrate) are classical PPAR‐α agonists that upregulate genes encoding CPT‐1A, acyl‐CoA oxidase, and mitochondrial medium‐chain acyl‐CoA dehydrogenase. They promote FAO in hepatocytes, decreasing hepatic TG accumulation [86]. However, clinical trials have shown modest efficacy in MASH resolution and inconsistent impact on fibrosis, and concerns exist regarding renal side effects [7, 87, 88]. On the other hand, elafibranor, a dual PPAR‐α/δ agonist, showed initial promise in improving insulin sensitivity and reducing steatosis in phase II trials. However, it failed to meet histological endpoints in the RESOLVE‐IT phase III trial (NCT02704403), curtailing its further development. Recently, lanifibranor, a pan‐PPAR agonist (PPAR‐α/γ/δ), has shown promise in improving steatosis, inflammation, and fibrosis in early studies (NCT03459079), and is currently undergoing further evaluation. This study has important clinical implications because it shows that targeting the core metabolic problems in MASLD (such as IR, lipotoxicity, and hyperglycemia) can enhance cardiometabolic health. It offers strong support for using lanifibranor to treat people with MASLD, either alone or combined with weight loss and other measures [89].

##### 5.2.2.3. SGLT‐2 Inhibitors

Sodium‐glucose cotransporter 2 (SGLT‐2) inhibitors are currently approved for the treatment of T2D. In addition to their blood glucose‐lowering efficacy, they also offer urinary and cardiovascular protection. SGLT‐2 inhibitors induce renal glucosuria, blood pressure reduction, and weight loss, with reductions in visceral, abdominal, and subcutaneous adipose tissue [90]. Due to these effects, SGLT‐2 inhibitors may be suitable for preventing or mitigating the development and progression of MASLD [83].

Mechanisms such as the reduction of inflammatory markers, the decrease in oxidative stress, and the decrease in hepatic lipogenesis influence the improvement in liver function after treatment with SGLT‐2 inhibitors [83]. The beneficial effects of these drugs in MASLD could be centered on reducing ER stress, improving mitochondrial function, and regulating autophagy and apoptosis [91–93].

##### 5.2.2.4. Biguanides Hypoglycemic Drugs

Biguanides are a class of antidiabetic drugs used for T2D. Metformin (dimethylbiguanide) is the most commonly prescribed drug for this treatment because of its glucose‐lowering effects, safety profile, and low cost. The primary target of biguanides is the mitochondrial complex I of the ETC [94, 95]. These drugs exhibit an inhibitory effect on complex I and inhibit the rate of oxygen consumption, leading to energy stress, an increased AMP/ATP ratio, and activation of AMPK, considered a key mediator of the therapeutic effects [96, 97]. Although metformin is not approved for the treatment of MASLD, combination therapy with other drugs has been documented to improve liver parameters. For example, a synergistic effect of SGLT‐2 inhibitors and metformin has been reported on hepatic and non‐hepatic complications in patients with T2D and MASLD [98].

#### 5.2.3. Other Molecules With Potential Benefits for MASLD

##### 5.2.3.1. Mitochondria‐Targeted Antioxidants (MTAs)

Unlike systemic antioxidants, MTAs selectively accumulate within the mitochondrial matrix and directly neutralize ROS, thus offering superior efficacy [99, 100]. Mitoquinone (MitoQ) is a coenzyme Q10 analog linked to a lipophilic triphenylphosphonium cation. MitoQ localizes selectively to the mitochondrial inner membrane. It mitigates oxidative stress by recycling ROS, improving mitochondrial membrane potential, and respiration. Preclinical studies show improvements in HFD‐induced steatosis, mitochondrial swelling, and lipid peroxidation [55, 101]. On the other hand, SkQ1 and SS‐31 (Elamipretide) are MTAs that stabilize mitochondrial membranes and inhibit lipid peroxidation. SS‐31 in particular binds cardiolipin, stabilizing mitochondrial cristae and enhancing ATP production. It also inhibits ROS formation at complexes I and III. In animal models, SS‐31 improved mitochondrial dynamics and decreased lipid peroxidation, although its clinical impact in liver disease remains underexplored [102]. However, SkQ1 is a plastoquinone‐based antioxidant that protects against mitochondrial ROS and apoptosis [103]. Preclinical hepatic studies are promising but require further validation in humans. Long‐term safety and efficacy in MASLD patients remain to be validated in large‐scale trials.

##### 5.2.3.2. Synthetic SIRT1s Activators

Sirtuins (SIRTs) are a family of highly conserved enzymes involved in a wide range of biological processes. They are located in the nucleus/nucleolus, mitochondria, and cytosol and can be classified into four distinct classes: class‐I (SIRT1‐SIRT3), class‐II (SIRT4), class‐III (SIRT5), and class‐IV (SIRT6 and SIRT7) [104]. SIRT1 is a prominent member of this family, which detects fluctuations in nicotinamide adenine dinucleotide (NAD^+^) levels and is involved in mitochondrial quality control and antioxidant defense [105]. The first generation of synthetic SIRT1 activators is derived from the imidazo[1,2‐b]thiazole core structure (SRT1720, SRT2183, and SRT1460), while the second generation of synthetic SIRT1 activators was developed through the optimization of the original imidazo[1,2‐b] thiazole scaffold structure (SRT2104 and SRT3025) [106]. Phase 2 clinical trials for SRT2104 (NCT01018017, NCT00937326, EUCTR2009‐010720‐26‐GB, EUCTR2009‐016537‐98‐DE) are being conducted to investigate the potential therapeutic applications in T2D; However, there is currently no evidence from studies in patients with MASLD.

##### 5.2.3.3. DNL Inhibitors

Targets associated with aberrant lipid metabolism represent a potential therapeutic strategy for MASLD. Inhibition of essential enzymes of DNL, such as ACC or fatty acid synthase (FAS), represents an attractive therapeutic approach [107–109]. For example, the ACC inhibitor GS‐0976 (NCT02856555) and FAS inhibitor TVB‐2640 (NCT04906421, NCT03938246) have demonstrated efficacy in Phase 2 clinical trials by improving hepatic steatosis and inhibiting hepatic DNL in patients with MASLD [5].

Other DNL inhibitors with different molecular targets have also been evaluated in clinical trials, demonstrating their ability to modulate the lipogenic pathway, either directly or indirectly. However, these are currently relatively small clinical studies, lasting less than 1 year. Therefore, larger and longer clinical trials will contribute to providing more robust evidence on the safety of these agents, whether alone or in combination with other DNL inhibitors, or even with other therapies [110, 111].

## 6. Future Directions

Due to the multifaceted pathophysiology of MASLD, a single drug is unlikely to address all pathophysiological components, and therefore, single‐target therapy may not offer optimal efficacy. Combination therapy is an increasing trend in the management of MASLD, as it involves multiple biological targets. These complementary actions may provide patients with greater benefits (e.g., synergistic effects or improved tolerability) than any monotherapy, potentially leading to better outcomes [112, 113]. For example, some studies have indicated that combining GLP‐1RAs with other therapies such as GLP‐1RA + FGF‐21 analogs [114], GLP‐1RA + THR‐β agonists [115], GLP‐1RA + SGLT‐2 inhibitors [115], or GLP‐1RA + DNL inhibitors [116] would potentially have greater efficacy, and in some cases, improved safety profiles, compared to monotherapies in patients with MASLD. Even, it has even been suggested that combining some synthetic drugs with natural products could be beneficial for these patients [117].

Finally, innovative therapies such as mitochondrial transplantation have garnered significant interest in recent years. Although this therapy is still under development, it is a promising strategy for restoring cellular metabolism. A recent study reported that replacing the dysfunctional mitochondrial network could attenuate MASH progression in an experimental model. In this study, healthy mitochondria inhibited disease progression by breaking down lipids in liver cells and increasing SIRT1 activity to restore cellular function [118].

## 7. Conclusions

In the liver, impaired mitochondrial quality control is a key contributor to mitochondrial dysfunction, which sets off a cascade of detrimental effects, including oxidative stress, inflammation, lipid accumulation, and IR, that underline the pathogenesis of various liver diseases, including MASLD. When overwhelmed by excess FFAs, largely driven by hypercaloric diets, these organelles become impaired, leading to a cascade of pathophysiological consequences that underlie the progression of MASLD to MASH, fibrosis, and eventually cirrhosis. Evidence from a wide range of dietary animal models consistently reveals structural and functional mitochondrial deterioration in parallel with liver histopathology. These findings have validated the mitochondrial axis as both a marker of disease severity and a target for therapeutic intervention. Encouragingly, many of these mitochondrial defects appear to be reversible. There is evidence that both non‐pharmacological and pharmacological therapies are able to significantly improve mitochondrial function. However, translating mitochondrial‐focused therapies into consistent clinical outcomes remains a challenge. Preserving or restoring mitochondrial integrity may represent a fundamental strategy in reversing the burden of MASLD. Bridging the gap between experimental evidence and clinical application through targeted, multi‐modal interventions holds great potential for transforming MASLD therapy in the short term.

## Author Contributions

Elda Cristina Villaseñor‐Tapia and Edgar Rubén Mendieta‐Condado: Writing–original draft; David Alejandro Curiel‐Pedraza: Writing–review and editing; Edwin Estefan Reza‐Zaldívar and Ana Laura Márquez‐Aguirre: conceptualization, writing–original draft, investigation, and formal analysis.

## Funding

No funding was received for this manuscript.

## Disclosure

All authors have read and agreed to the published version of the manuscript.

## Conflicts of Interest

The authors declare no conflicts of interest.

## Data Availability

Data sharing is not applicable to this article as no datasets were generated or analyzed during the current study.
